# Prevalence and associated risk factors of chronic bronchitis in First Nations people

**DOI:** 10.1186/s12890-017-0432-4

**Published:** 2017-06-29

**Authors:** Punam Pahwa, Chandima P. Karunanayake, Donna C. Rennie, Joshua A. Lawson, Vivian R. Ramsden, Kathleen McMullin, P. Jenny Gardipy, Judy MacDonald, Sylvia Abonyi, Jo-Ann Episkenew, James A. Dosman, the First Nations Lung Health Project Research Team

**Affiliations:** 1 Canadian Centre for Health and Safety in Agriculture, Royal University Hospital, 104, Clinic Place, Saskatoon, SK S7N 2Z4 Canada; 2 Department of Community Health and Epidemiology, University of Saskatchewan, 104 Clinic Place, Saskatoon, S7N 2Z4 SK Canada; 3 Department of Academic Family Medicine, University of Saskatchewan, West Winds Primary Health Centre, 3311 Fairlight Drive, Saskatoon, S7M 3Y5 SK Canada; 4 Community A, Saskatchewan, Canada; 5 Community B, Saskatchewan, Canada; 6 Deceased, Former Faculty of Indigenous Peoples’ Health Research Centre, University of Regina, Saskatchewan, Canada

**Keywords:** First Nations, Chronic bronchitis, Environmental tobacco smoke, Mould or mildew

## Abstract

**Background:**

Inadequate housing, low family income, household smoking, personal smoking status, and poor schooling are some of the conditions that have been significantly associated with the prevalence and incidence of chronic bronchitis. The aim of the current study was to determine the prevalence of chronic bronchitis (CB) and associated risk factors among First Nations people.

**Methods:**

An interviewer-administered survey was conducted as part of the First Nations Lung Health Project in 2012 and 2013 with 874 individuals from 406 households in two First Nations communities located in the province of Saskatchewan, Canada. The questionnaire collected information on individual and contextual determinants of health and a history of ever diagnosed with CB (outcome variable) from the two communities participating in the First Nations Lung Health Project. Clustering effect within households was adjusted using Generalized Estimating Equations.

**Results:**

The prevalence of CB was 8.9% and 6.8% among residents (18 years and older) of community A and community B respectively and was not significantly different. CB prevalence was positively associated with odour or musty smell of mildew/mould in the house [OR_*adj*_ (95% CI) = 2.33 (1.21, 4.50)], allergy to house dust [3.49 (1.75, 6.97)], an air conditioner in home [2.33 (1.18, 4.24)], and increasing age [0.99 (0.33, 2.95), 4.26 (1.74, 10.41), 6.08 (2.58, 14.33)]. An interaction exposure to environmental tobacco smoke in the house*body mass index showed that exposure to household smoke increased the risk of CB for overweight and obese participants (borderline). Some of the variables of interest were not significantly associated with the prevalence of CB in multivariable analysis, possibly due to small numbers.

**Conclusions:**

Our results suggest that significant determinants of CB were: increasing age; odour or musty smell of mildew/mould in the house; allergy to house dust; and, body mass index. Modifiable risk factors identified were: (i) community level-housing conditions (such as mould or mildew in home, exposure to environmental tobacco smoke in house); and, (ii) policy level-remediation of mould, and obesity.

**Trial registration:**

Not applicable.

## Background

The overall respiratory health of Aboriginal peoples (First Nations, Metis, and Inuit) is poorer than that of the general Canadian population [1, 2]. Rates of respiratory diseases including asthma, chronic bronchitis (CB), and chronic obstructive pulmonary disease (COPD) are higher in Aboriginal peoples [1, 3]. According to the First Nations Regional Longitudinal Health Survey, in 2002/03 the age-standardized prevalence of self-reported, physician-diagnosed CB was 3.7% in First Nations people living on reserves [4]; and according to the Canadian Community Health Survey (CCHS) 2005, the prevalence of CB in Aboriginal people living off-reserve was 4.9% [5]. In a recently published article, based on data from the 2006 Aboriginal Peoples Survey (APS), the prevalence of CB was 6.6% among off-reserve First Nations people (5.0% in males and 7.2% in females) [2]. These rates were higher than the prevalence of CB for the non-Aboriginal Canadian population (2.4%) [5]. CB is a respiratory disease defined as “*cough productive of sputum for at least 3 months of the year for at least 2 years*” [6]. Research has shown that CB is a significant cause of morbidity and an underlying condition for the development of COPD [7]. Based on limited data available for on-reserve First Nations, it was reported that First Nations adults living on reserves have higher age-adjusted rates of CB compared to other Canadians [8, 9].

Exposure to cigarette smoke, inadequate housing, low socio-economic status, and obesity are some of the factors associated with the increased prevalence and incidence of CB [5, 10, 11]. These factors are more prevalent among First Nations people as compared to the general Canadian population and might be responsible for the high prevalence of CB in Aboriginal peoples [1, 9, 12–15]. Approximately 11% of Canada’s Aboriginal peoples live in Saskatchewan [16]. To date, knowledge about the risk factors associated with CB among on-reserve First Nations peoples is limited and has not been well established. The purpose of the baseline component of First Nations Lung Health Project (FNLHP) conducted in 2012 was to address these gaps. The objective of this manuscript was to compute the prevalence rates of CB and determine its associated risk factors in on-reserve First Nations people.

## Methods

The FNLHP is a prospective cohort study being conducted using interviewer-administered surveys in two phases, the baseline, and the follow-up. The baseline survey was completed between 2012 and 2013. The details of this study are given elsewhere [17]. In this study, through the application of a Population Health Framework, we attempt to understand the association between the individual and contextual factors; as well as the interactions between them in relation to respiratory health outcomes after adjusting for important covariates [18]. In brief, two on-reserve communities were invited to participate in this study. Henceforth, we will call these communities Community A and Community B. These communities were selected based on a long-term previously established relationships. The questionnaire collected information on individual and contextual factors and a history of ever diagnosed with CB (outcome variable). Some measures of housing conditions, life-style and socio-economic status used in our questionnaires were adopted from previous research studies that had validated these measures [18, 19].

### Primary respiratory health outcome

The primary outcome of interest was self-reported physician-diagnosed CB, as determined from the baseline survey question: “Has a doctor ever said you had … Chronic Bronchitis?”.

### Contextual factors

The primary contextual factors associated with respiratory health outcomes were crowding (based on a ratio of number of people who usually live in the household and number of bedrooms), socioeconomic status (assessed using total household income), indoor air quality (assessed by response to questions about the quality of house—in need of major/minor repairs, water or dampness, damage caused by dampness, mildew odor or musty smell, mold or mildew, presence of proper ventilation such as the use of an air conditioner, humidifier or dehumidifier, pets inside home and environmental tobacco smoke in the house), and outdoor environment (assessed by responses to questions about having an outdoor corral or feedlot, bales, grain bins, sewage pond or manure lagoon, garbage dump, lumber yard or saw mill near the home).

### Individual factors

The primary individual factors considered were the highest level of educational attainment, lifestyle and behavioral factors inclusive of smoking, physical activity, and allergies.

### Covariates

Covariates included in this analysis were age, sex, and body mass index (BMI).

### Statistical analysis

The percentage of respondents (overall and separately for males, females) self-reporting doctor diagnosed CB was calculated for each community. The percentages of respondents for each risk factor and the self-reported doctor diagnosed CB were also calculated. The bi-variable analysis was used to select variables for multivariable logistic regression based on the standard model building techniques [20]. A multivariable logistic regression modeling technique based on maximum likelihood was used to test the association between risk factors and the presence of chronic bronchitis. Clustering effects within households were adjusted using Generalized Estimating Equations. The strength of association was presented as odds ratio (ORs) estimates and 95% confidence intervals (CIs). SPSS 22 was used to conduct all analyses [21].

## Results

The FNLHP included 874 adults participants (response rate: 55.7% of 1570 based on 2011 Canadian Census) (443 women, 431 men) living in 406 households (response rate: 70% of 580 based on band lists) from the two participating communities. This analysis was based on *n* = 720 participants who responded to the question “Has a doctor ever said you had chronic bronchitis?” and completed an individual and household questionnaires.

**Fig. 1:**
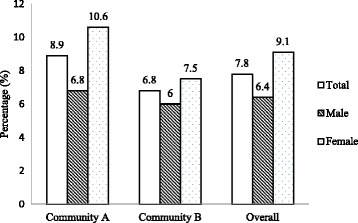
Prevalence of chronic bronchitis in two communities (overall and by sex)

**Fig. 2:**
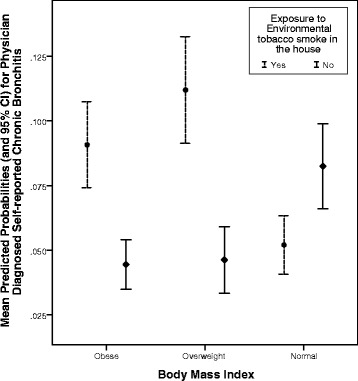
Mean predicted probabilities for the interaction between body mass index and exposure to environmental tobacco smoke in the house

The mean age of the study population was 34.83 ± 14.47 years and 17.8% were older than 50 years. The demographics of the population were: 52.1% females; 28.8% overweight; 35.4% obese; 76.8% current smokers; and, 13.5% ex-smokers. The overall prevalence of CB was 7.8% (Fig. 1). Prevalence of CB in Community A was 8.9% (6.8% in males and 10.6% in females) and in Community B was 6.8% (6.0% in males and 7.5% in females) (Fig. 1). In both communities, the prevalence of CB in females was higher compared to the prevalence of CB in males but not statistically significant (Community A - *p* value = 0.21; Community B - *p* value = 0.57). The univariate relationships between the contextual factors and individual factors or covariates and CB using unadjusted logistic regression are shown in Table 1. The multivariable logistic regression results are presented in Table 2. The significant predictors of CB were: increasing age; the presence of mildew odor or musty smell in the house; having an air conditioner; and, having a reported allergic reaction to house dust. Exposure to environmental tobacco smoke in the house modified the relationship between BMI and chronic bronchitis (*p* < 0.05). That is, a person who was obese or overweight and exposed to environmental tobacco smoke in the house had an increased risk of self-reported, doctor-diagnosed CB compared to a person who was not exposed to environmental tobacco smoke in the house (Fig. 2).

**Table 1:** Bi-variable analysis of the association of chronic bronchitis on personal and environmental factors (*n* = 720)

	Ever Diagnosed with Chronic Bronchitis		Unadjusted^a^ Odds Ratio (95% CI)
	Yes/Total	%	
Contextual Factors			
Socioeconomic			
Annual income			
No income	2/42	4.8	0.73 (0.16, 3.45)
$0–$9999	16/151	10.6	1.71 (0.77, 3.78)
$10,000–$19,999	10/110	9.1	1.42 (0.65, 3.11)
Unknown/Not stated	15/216	6.9	1.07 (0.49, 2.33)
≥ $20,000	13/201	6.5	1.00
Crowding			
> 1 person/bedroom	40/509	7.9	1.17 (0.39, 3.53)
= 1 person/bedroom	11/137	8.0	1.20 (0.35, 4.15)
< 1 person/bedroom	5/74	6.8	1.00
Indoor Environmental Factors			
Environmental Tobacco Smoke (Household Smoking)			
Yes	37/405	9.1	1.56 (0.87, 2.79)
No	19/312	6.1	1.00
House in need of repairs			
Yes, major repairs	23/289	8.0	1.22 (0.61, 2.45)
Yes, minor repairs	18/198	9.1	1.39 (0.65, 2.98)
No, only regular maintenance	14/211	6.6	1.00
During past 12 months, water or dampness			
Yes	33/433	7.6	0.83 (0.47, 1.48)
No	22/244	9.0	1.00
House damages caused by dampness			
Yes	33/374	8.8	1.51 (0.83, 2.72)
No	18/299	6.0	1.00
Mildew odor or musty smell in home			
Yes	38/369	10.3	**2.18 (1.18, 4.03)**
No	15/300	5.0	1.00
Signs of mold or mildew in home			
Yes	33/336	9.8	1.75 (0.95, 3.23)
No	18/306	5.9	1.00
Wood store or wood to heat house			
Yes	1/27	3.7	0.46 (0.06, 3.59)
No	55/693	7.9	1.00
House heating system has a filter			
Yes No	50/560 6/160	8.9 3.8	**2.50 (1.06, 5.88)** 1.00
House has an air conditioning			
Yes	23/186	12.4	**2.14 (1.22, 3.75)**
No	33/534	6.2	1.00
Humidifier used in house			
Yes	3/102	2.9	0.33 (0.10, 1.06)
No	53/618	8.6	1.00
Dehumidifier used in house			
Yes	7/73	9.6	1.30 (0.48, 3.51)
No	49/647	7.8	1.00
In the past 12 months, pet living in house			
Cat			
Yes	8/109	7.3	0.93 (0.41, 2.11)
No	48/611	7.9	1.00
Dog			
Yes	11/167	6.6	0.79 (0.39, 1.61)
No	45/553	8.1	1.00
Bird			
Yes	5/31	16.1	2.41 (0.83, 6.96)
No	51/689	7.4	1.00
Other			
Yes	2/32	6.3	0.80 (0.18, 3.54)
No	54/688	7.9	1.00
Outdoor Environment near your home			
An outdoor corral or feedlot			
Yes	13/120	10.8	1.56 (0.80, 3.05)
No	43/600	7.2	1.00
A bales stack or bales			
Yes	9/102	8.8	1.14 (0.52, 2.53)
No	47/618	7.6	1.00
Grain bins			
Yes	5/44	11.4	1.55 (0.54, 4.41)
No	51/625	7.5	1.00
Sewage pond or manure lagoon			
Yes	20/202	9.9	1.45 (0.82, 2.59)
No	36/518	7.0	1.00
Garbage dump			
Yes	14/144	9.7	1.36 (0.73, 2.57)
No	42/576	7.3	1.00
Lumber yard, carpentry construction or sawmill			
Yes	2/34	5.9	0.71 (0.10, 5.21)
No	54/686	7.9	1.00
Individual Factors			
Smoking Status			
Current smoker	42/553	7.6	1.05 (0.42, 2.63)
Ex-smoker	9/97	9.3	1.32 (0.42, 4.16)
Never smoker	5/70	7.1	1.00
Education			
< High School	24/352	6.8	0.76 (0.42, 1.38)
≥ High School	32/366	8.7	1.00
Exercise			
Yes	39/498	7.8	1.03 (0.57, 1.85)
No	17/222	7.7	1.00
An allergic reaction to			
House dust			
Yes	20/100	20.0	**4.08 (2.31, 7.22)**
No	36/620	5.5	1.00
Cat			
Yes	7/70	10.0	1.37 (0.62, 3.02)
No	49/650	7.5	1.00
Dog			
Yes	2/23	8.7	1.15 (0.27, 4.94)
No	54/697	7.7	1.00
Grasses, pollens or trees			
Yes	13/88	14.8	**2.37 (1.21, 4.63)**
No	43/632	6.8	1.00
Molds			
Yes	18/88	20.4	**4.08 (2.18, 7.62)**
No	38/632	6.0	1.00
Farm Animals			
Yes	1/18	5.6	0.70 (0.09, 5.28)
No	55/702	7.8	1.00
Tobacco smoke			
Yes	10/61	16.4	**2.62 (1.28, 5.36)**
No	46/659	7.0	1.00
Covariates			
Age, in years			
> 50	21/128	16.4	**5.94 (2.61, 13.52)**
36–50	21/162	13.0	**4.56 (1.99, 10.41)**
26–35	6/174	3.5	1.08 (0.40, 2.95)
18–25	8/256	3.1	1.00
Sex			
Female	34/375	9.1	1.47 (0.84, 2.57)
Male	22/345	6.4	1.00
Body Mass Index (kg/m^2^)			
Obese (>30)	19/255	7.5	1.21 (0.59, 2.45)
Overweight (25–30)	18/207	8.7	1.43 (0.71, 2.88)
Normal (0− < 25)	15/240	6.2	1.00

Within household clustering is accounted for by multi-level univariate

^a^logistic regression; odds ratios that are significantly different from 1.00 (*p* < 0.05) are in bold face

**Table 2:** Odds Ratios (95% confidence intervals) based on multivariate logistic regression^a^ for associations with chronic bronchitis

Variable	Adjusted^b^ OR (95% CI)
Household smoking	
Yes	0.36 (0.11, 1.14)
No	1.00
Mildew odour or musty smell in home	
Yes	**2.33 (1.21, 4.50)**
No	1.00
House has an air conditioner	
Yes	**2.23 (1.18, 4.24)**
No	1.00
Age, in years	
> 50	**6.08 (2.58, 14.33)**
36–50	**4.26 (1.74, 10.41)**
26–35	0.99 (0.33, 2.95)
18–25	1.00
Sex	
Female	1.49 (0.76, 2.92)
Male	1.00
Body Mass Index (kg/m^2^)	
Obese (>30)	0.17 (0.05, 0.59)
Overweight (25–30)	0.19 (0.06, 0.63)
Normal (0− < 25)	1.00
Allergy to house dust	
Yes	**3.49 (1.75, 6.97)**
No	1.00
Interaction (See Fig. 1)	*P* value
Household smoking X Body Mass Index	**<0.05**

^a^Adjusted for repeated measure on households

^b^logistic regression; odds ratios that are significantly different from 1.00 (*p* < 0.05) are in bold face

A sub-analysis was completed to explore the 2-way interaction between sex and smoking (in terms of pack-years) and CB. Smoking pack-years, a continuous variable, was divided into three categories: 0 for never-smokers; > 0 and < 10 pack-years (moderate smokers); and ≥ 10 pack-years (heavy smokers). Based on crosstabs analysis, we observed (data not shown), for females there was a significant positive association between smoking pack-years and the prevalence of CB (5.4% self-reported, doctor-diagnosed CB among never-smokers, 4.3% among ex-smokers, and 21.3% among current-smokers); however, this relationship was not significant for males. The association between smoking pack-years and prevalence of CB was different for males and females, indicating that sex is an effect modifier in the relationship between smoking pack-years and the prevalence of CB. However, we were not able to test this interaction in the multivariable model because of relatively small numbers due to pack-years information missing for approximately 40% of the study population.

## Discussion

By using the information from the baseline component of the longitudinal study, we determined the prevalence of CB and examined the associated risk factors in First Nations people residing in two on-reserve communities in Saskatchewan. In this study, a fairly good response for household participation for completing the baseline survey via interviewer-administered questionnaire was observed (53.9% in Community A and 89.9% in Community B). The overall prevalence of CB was 7.8% in these two on-reserve First Nations communities (6.4% in males and 9.1% in females). Modifiable risk factors identified were housing conditions such as mildew odor or musty smell in home, having an air conditioner in the home, exposure to environmental tobacco smoke in the house and obesity in the communities. The other significant risk factors of CB were increasing age and allergic reaction to house dust.

Geographic location (reserve) appears to have a large impact with respect to health status and use of physician services [22–24]. The prevalence of on-reserve First Nations peoples (7.8%, based on the current study) was higher than the off-reserves Aboriginals (6.6% based on the 2006 APS [2] and 4.9% based on the 2005 CCHS [5]). In contrast, the 2002/03 First Nations Regional Longitudinal Health Survey reported that age-standardized prevalence of self-reported, doctor-diagnosed CB was 3.7% in First Nations living on-reserve [4]. All these rates are higher than the prevalence of 2.4 and 2.5% found in the non-Aboriginal Canadian population, according to the 2005 CCHS and 2007/2008 CCHS, respectively [5, 25]. A recent study of Saskatchewan rural residents (excluding Aboriginal residents) reported a prevalence of 5.9% [26]. These differences in the prevalence of CB support the findings that geographical location (on-reserve, off-reserve and rural) has a large impact on respiratory health.

An article based on the 2006 APS including off-reserve First Nations, Metis, and Inuit people observed that older age, lower educational attainment, lower income, and urban residence were significantly associated with self-reported doctor-diagnosed CB [2]. It was also reported that smoking status and body mass index were significantly associated with CB, and their effects differed by sex [2]. A recent Saskatchewan Rural Health Study (SRHS) [26] reported a positive association between greater prevalence of CB and lower household income adequacy, increasing age, allergies, history of lung disease in a parent, exposure to stubble smoke, obesity, prenatal exposure to smoking, and female sex. The SRHS also reported that smoking modified the relationship of CB with occupational exposure to wood dust, solvents and allergic reaction to solvents [26]. Similar to these studies, the current study reported that older age was significantly associated with CB. In contrast, based on our data we did not observe any association between education level, income level, and the prevalence of CB. This could be due to small numbers and less variation in the data related to income and education. In this study, approximately 27% of households reported an annual income of < $10,000 or no income, and, 15% reported being in the $10,000–$19,999 annual income range. Thirty percent of households did not report the income. About half of them had less than a high school education. For the current study, we did not collect information on a history of lung diseases in a parent, exposure to stubble smoke, and prenatal exposure to smoking. There was a positive observable association between allergies and CB when we conducted univariate analysis, but the association was not significant when multivariate analysis was conducted.

Exposure to environmental tobacco smoke (household smoke) is the most important risk factor for the development of CB as reported by several studies [2, 25–29]. The current study did not report a significant association between personal non-traditional use of tobacco and the prevalence of CB; however, there was a significant interaction with environmental tobacco smoke and BMI. In the participating two on-reserve communities, 77% were currently engaged in the non-traditional use of tobacco, 13% had been ex-smokers and only 6% of smoke-free individuals implying that almost everyone in the household had been exposed to secondhand smoke. Hence, we decided to investigate the association between household smoke and CB. It is possible that, due to the high proportion of those who currently or have in the past engaged in the non-traditional use of tobacco, we did not find any positive association between non-traditional use of tobacco and CB in spite of a vast literature available on the positive association between smoking and CB. Data from the CCHS 2000/2001 reported that self-reported recent secondhand smoke exposure in Canada was high and was associated with asthma, chronic bronchitis, and hypertension in never- and ex-smokers [30]. Few other studies reported the relationship between secondhand smoke and CB [31, 32].

In our study, the prevalence of CB was 6.4% for males and 9.1% for females, supporting the earlier finding of higher prevalence of CB in females in Aboriginal and rural populations [2, 26]. Based on our recent study of small town and rural people in Saskatchewan, we observed that females were significantly more likely to self-report doctor-diagnosed CB (OR 1.32, 95% CI: 1.10–1.58). In our study, the proportion of current smokers (78.5% vs. 76.0%) were higher for males compared to females, and there were higher proportions of ex-smokers (14.4% vs. 12.1%) among females compared to males. Research has shown a strong positive association between smoking and CB and a higher prevalence of CB in females [2, 25–28]. A univariate sub-analysis of this study revealed that the association between smoking pack-years and prevalence of CB was different for males and females, indicating that sex is an effect modifier in the relationship between smoking pack-years and the prevalence of CB.

Research has shown that environmental tobacco smoke contains more than 4000 chemicals and many of these are potent respiratory irritants [33]. These chemicals pollute the environment and damage human lungs when inhaled [33]. In the current study, BMI modified the relationship between exposure to secondhand smoke in the house and the prevalence of CB. Overweight and obese individuals who were exposed to secondhand smoke in the house were more likely to self-report doctor-diagnosed CB compared to those who were not exposed to secondhand smoke in the house.

An intergenerational link has been shown between attendance at a residential school, smoking, and CB [4, 34]. We explored the association between “Have you ever attended the residential school?” and “Have either of your parents/grandparents attended the residential school?” and the prevalence of CB. We did not find a significant association between either of the variables related to residential school and the prevalence of CB.

### Strengths and limitations

One of the strengths of our study was the moderate response rates for household surveys and individual surveys. Very few Canadian studies have examined the risk factors of chronic bronchitis among First Nations peoples. In general, due to the cross-sectional nature of the study, one of the major limitations was the recall-bias of disease history. Another limitation is that the information bias due to hiring local people to conduct interviews.

## Conclusions

The prevalence of CB in the First Nations people could be high because risk factors associated with the prevalence of CB are more common in these communities compared to the general Canadian population. CB occurred as a result of both non-modifiable (such as age) and modifiable risk factors. Modifiable risk factors identified were housing conditions such as mildew odor or musty smell in the home, secondhand smoke in the house, and obesity. We observed interaction effects on prevalence of CB between secondhand smoke in the house and BMI (adjusting for other variables in the model) and smoking pack-years and sex (not adjusting for any other variable) for First Nations peoples. These interaction effects have not been widely reported previously about on-reserve First Nations communities. We have established a baseline for the prevalence of CB among First Nations peoples in Saskatchewan against which future research related to the control of this disease can be developed.

## Abbreviations

APS: Aboriginal peoples survey
BMI: Body mass index
CB: Chronic bronchitis
CCHS: Canadian Community Health Survey
CI: Confidence interval
COPD: Chronic obstructive pulmonary disease
FNLHP: First Nations Lung Health Project
SPSS: Statistical Package for the Social Science
SRHS: Saskatchewan Rural Health Study

## References

[1] Sin DD, Wells H, Svenson LW, Man SF (2002). Asthma and COPD among Aboriginals in Alberta, Canada. Chest.

[2] Konrad S, Hossain A, Senthilselvan A, Dosman JA, Pahwa P (2013). Chronic bronchitis in Aboriginal people—prevalence and associated factors. Chronic Dis Injuries Can.

[3] Health Canada. A statistical profile on the health of First Nations in Canada: Determinants of Health 1999–2003. Ministry of Health Publication No: 3555; Cat No: H34-193/1-2008, 2009.

[4] First Nations Information Governance Committee (2005). First Nations Regional Longitudinal Health Survey (RHS) 2002–03; Results for Adults, Youth and Children Living in First Nations Communities.

[5] Minore B, Hill ME, Park J (2010). Understanding respiratory conditions among Ontario’s Aboriginal population.

[6] American Thoracic Society (1962). Definitions and classifications of chronic bronchitis, asthma and pulmonary emphysema: a statement by the Committee on Diagnostic Standards for Nontuberculous Respiratory Diseases. Am Rev Respir Dis.

[7] Pelkonen M (2008). Smoking: Relationship to chronic bronchitis, chronic obstructive pulmonary disease and mortality. Curr Opin Pulm Med.

[8] Earle L (2011). Understanding chronic disease and the role of traditional approaches in Aboriginal communities.

[9] Butler-Jones D (2008). The chief public health officer’s report on the state of public health in Canada addressing health inequalities.

[10] Melia RJW, Chinn S, Rona RJ (1988). Respiratory illness and home environment of ethnic groups. Br Med J.

[11] Cooreman J, Redon S, Levallois M, Liard R, Perdrizet S (1990). Respiratory history during infancy and childhood, and respiratory conditions in adulthood. Int J Epidemiol.

[12] Wong S (2006). Use and misuse of tobacco among Aboriginal peoples. Paediatr Child Health.

[13] Aboriginal statistics at a glance (2016). Income [Internet].

[14] Educational Portrait Of Canada (2006). Census [Archived].

[15] Aboriginal Peoples in Canada in 2006: Inuit, Metis and First Nations, 2006 Census [Internet]. Ottawa (ON): Statistics Canada; 2016. http://www12.statcan.ca/census-recensement/2006/as-sa/97-558/pdf/97-558-XIE2006001.pdf. Accessed on 20 July 2016.

[16] Aboriginal peoples in Canada: First Nations People, Metis and Inuit. Ottawa (ON): Statistics Canada; 2016. http://www12.statcan.gc.ca/nhs-enm/2011/as-sa/99-011-x/99-011-x2011001-eng.cfm. Accessed on 20 July 2016.

[17] Pahwa P, Abonyi S, Karunanayake C (2015). The First Nations Lung Health Project: Assessing, Redressing, and Re-assessing Disparities in Respiratory Health among First Nations People. BMC Res Notes.

[18] Canada H (1994). Strategies for Population Health: Investing in the Health of Canadians. Cat. No. H39-316/1994E.

[19] Aboriginal Peoples Survey (APS), 2006 [Internet]. Ottawa (ON): Statistics Canada; 2016. http://www5.statcan.gc.ca/olc-cel/olc.action?objId=89-637-X&objType=2&lang=en&limit=0. Accessed on 22 July 2016.

[20] Hosmer DW, Lemeshow S (2013). Applied Logistic Regression.

[21] IBM Corp (2013). Released 2013. IBM SPSS Statistics for Windows, Version 22.0.

[22] Newbold KB (1998). Problems in search of solutions: health and Canadian aboriginals. J Community Health.

[23] Elliott SJ, Foster LT, Stephensen PH, Elliott SJ (1995). Mind-Body-Place: a geography of Aboriginal health in British Columbia. A Persistent Sprit: Towards An Understanding of Aboriginal Health in British Columbia.

[24] National Collaborating Centre for Aboriginal Health (2011). Access to Health Services as a social determinant of First Nations, Inuit and Metis Health.

[25] Karunanayake CP, Hagen B, Dosman JA, Pahwa P (2013). Prevalence and risk factors of chronic bronchitis in a Canadian population: the Canadian Community Health Survey, 2007–2008. Can Respir J.

[26] Pahwa P, Karunanayake C, Willson PJ (2012). Prevalence of Chronic Bronchitis in Farming and Non-Farming Rural Residents in Saskatchewan. J Occup Environ Med.

[27] Snider GL (1985). Distinguishing among asthma, chronic bronchitis, and emphysema. Chest.

[28] Troisi RJ, Speizer FE, Rosner B, Trichopoulos D, Willett WC (1995). Cigarette smoking and incidence of chronic bronchitis and asthma in women. Chest.

[29] Cohen J, Powderly W, Opal S. Infectious Diseases, 3nd ed. Philadelphia: Mosby (Elsevier); 2010. Chapter 26: Bronchitis, Bronchiectasis, and Cystic Fibrosis. p. 276–83.

[30] Vozoris N, Lougheed MD (2008). Second-hand smoke exposure in Canada: Prevalence, risk factors, and association with respiratory and cardiovascular disease. Can Respir J.

[31] Leuenberger P, Schwartz J, Ackermann-Liebrich U (1994). Passive smoking exposure in adults and chronic respiratory symptoms (SAPALDIA Study). Swiss Study on Air Pollution and Lung Diseases in Adults, SAPALDIA Team. Am J Respir Crit Care Med.

[32] Simoni M, Baldacci S, Puntoni R (2007). Respiratory symptoms/diseases and environmental tobacco smoke (ETS) in never smoker Italian women. Respir Med.

[33] The Lung Association-Your Healthy Home. Fact Sheet: Second hand smoke. 2016. https://www.on.lung.ca/document.doc?id=1570. Accessed on 5 Aug 2016.

[34] Smith D, Varcoe C, Edwards N (2005). Turning around the intergenerational impact of residential schools on Aboriginal people: implications for health policy and practices. Can J Nurs Res.

